# CHX and a Face Shield Cannot Prevent Contamination of Surgical Masks

**DOI:** 10.3389/fmed.2022.896308

**Published:** 2022-05-23

**Authors:** Madline P. Gund, Jusef Naim, Matthias Hannig, Alexander Halfmann, Barbara Gärtner, Gabor Boros, Stefan Rupf

**Affiliations:** ^1^Department of Operative Dentistry, Periodontology and Preventive Dentistry, Saarland University, Homburg, Germany; ^2^Oral Surgery Clinic, German Armed Forces Central Hospital, Koblenz, Germany; ^3^Institute of Medical Microbiology and Hygiene, Saarland University, Homburg, Germany; ^4^Chair of Synoptic Dentistry, Universität Des Saarlandes, Homburg, Germany

**Keywords:** infection control, COVID-19, surgical mask, aerosols, microbiology, dentistry, face shield, chx

## Abstract

**Background:**

Bacterial contamination on surgical masks puts a threat to medical staff and patients. The aim of the study was to investigate its contamination during dental treatments, wearing a face shield and performing a pre-procedural mouth rinsing with chlorhexidine (CHX).

**Methods:**

In this prospective, randomized study, 306 treatments were included, 141 single-tooth (restorations) and 165 total dentition treatments (preventive or periodontal supportive ultrasonic application). A total of three groups (each: *n* = 102) were formed: participants rinsed for 60 s with 0.1 % CHX or with water before treatment, and, for control, a non-rinsing group was included. In view of the COVID-19 pandemic, a face shield covering the surgical mask enhanced personal protective equipment. After treatment, masks were imprinted on agar plates and incubated at 35°C for 48 h. Bacteria were classified by phenotypic characteristics, biochemical assay methods, and matrix-assisted laser desorption/ionization time of flight mass spectrometry (MALDI-TOF MS). Colonies (CFU) were counted and mean values were compared (Kruskal–Wallis-, *U* test, *p* < 0.05).

**Results:**

Chlorhexidine led to a statistically significant reduction of bacterial contamination of the surgical mask (mean: 24 CFU) in comparison with water (mean: 47 CFU) and non-rinsing (mean: 80 CFU). Furthermore, rinsing with water reduced CFU significantly in comparison with the non-rinsing group. There were no significant differences between single or total dentition treatments. *Streptococcus* spp., *Staphylococcus* spp., *Micrococcus* spp., and *Bacillus* spp. dominated, representing the oral and cutaneous flora.

**Conclusion:**

A pre-procedural mouth rinse is useful to reduce the bacterial load of the surgical mask. However, contamination cannot be prevented completely, even by applying a face shield. In particular, during pandemic, it is important to consider that these additional protective measures are not able to completely avoid the transmission of pathogens bearing aerosols to the facial region. If antiseptic rinsing solutions are not available, rinsing with water is also useful.

## Introduction

Surgical masks are contaminated during dental treatments (1, 2) and can be a source of further contamination itself (3). Recommendations have been made so far to prevent contamination pathways. These include changing the face mask regularly after each treatment and avoiding hand contact with the mask. In addition, the face mask should be discarded immediately after the treatment and should not be placed on the surfaces (3).

Sachdev et al. also investigated dental treatments and found significantly higher contamination on the outside of the mask than on the inside, pointing out that more attention needs to be paid to reduce microbiological contamination in the clinical environment in daily routine. In addition, protective measures should be taken to improve air quality (4). Luksamijarulkul et al. support these results, also finding a greater contamination on the outside of the mask than on the inside. This in turn correlates with samples from the air (5).

Chlorhexidine (CHX) rinsing is the antiseptic gold standard in everyday dental practice and can lower microbiological contamination. Retamal-Valdes et al. investigated the effect of various pre-procedural mouthwashes including a rinse containing cetylpyridinium chloride (CPC), zinc (Zn), sodium fluoride (F), CHX, water, and no rinse in ultrasonic treatments on the support board, patient's chest and dentist's forehead using agar plates. A significant reduction in pathogens was observed with CPC + Zn +F and CHX rinsing on the dentist's forehead, the patient's chest, and overall, with the forehead benefiting the most and the support board the least (6). Gupta et al. studied a pre-procedural CHX rinse in periodontal treatments and its effect on contamination of the practitioner's, assistant's, and patient's chest leading to a significant pathogen reduction in comparison with herbal mouthwashes and water (7). At the latest since the beginning of the COVID-19 pandemic, pre-procedural mouthwashes have become a focus of attention (8).

During the COVID-19 pandemic, greater awareness of aerosols possibly containing pathogenic microorganisms developed (9). The standardized personal protective equipment (PPE) for the dentist was therefore extended. Since infectious bioaerosols or droplets can contaminate the human conjunctival epithelium and COVID-19 can be transmitted through contact with mucous membranes, a face shield or respectively, a protective eyewear was additionally recommended for dentists and ophthalmologists (10, 11). Surgical masks continued to be worn for treatments <1 m apart. A FFP2 or KN95 mask was recommended for aerosol-producing procedures and a FFP3 mask for suspected patients with COVID-19 (11). Reviews provide full recommendations, which protective equipment dentists should wear, accompanied by instructions to remove the PPE to avoid cross-contaminations (12).

To our knowledge, it has not been investigated so far, whether a pre-procedural CHX rinse in combination with a face shield can reduce the contamination of the practitioner's surgical mask. Therefore, the aim of the study was to investigate the efficacy of a pre-procedural mouth rinse with CHX in comparison with water and no rinse on the bacterial load of the mask, always in combination with a face shield.

## Materials and Methods

### Setting

This single-center, prospective, randomized, three-group parallel design was conducted at Saarland University Dental Center. All instruments used were sterile. The dental unit and the surrounding surfaces were disinfected by wiping (Celtex® Wipes, Lotfex, Bremen, Germany; Incidin®, Dräger, Lübeck, Germany). The room temperature was 20–22°C with 40–60% relative humidity.

### Treatments

A total of three-hundred six aerosol-producing dental treatments were included: 141 single-tooth interventions consisting of high-speed preparation of a carious cavity and tooth substance for restorative purpose and 165 total dentition treatments consisting of a supra- and subgingival ultrasonic application during preventive or supportive periodontal measures. The duration of treatment was on average of 60–90 min. For all types of treatment, dental unit water was used for cooling. An evacuation was established by means of conventional dental suction (CDS) using a cannula of 3.3 mm in diameter (suction flow 1.1 l/s) and high-volume evacuation (HVE, tube of 8.0 mm in diameter, suction flow 6.0 l/s). The CDS was placed lingually from the lower central incisors. The HVE was held by an assistant near to the aerosol source.

### Patients

Only adults were included. Infectious diseases or antibiotic use in the last 6 months led to exclusion. Verbal informed consent was obtained from all participants. No individual patient or practitioners' data were recorded. All samples were anonymized. A total of three patient groups (each group: *n* = 102) were formed: the first group rinsed for 60 s with 15 ml 0.1% CHX (intervention group), the second for 60 s with 15 ml water (control group), the third group did not rinse (non-intervention) before treatment. Ethical approval for the study was obtained from the Ethics Committee of the Saarland Medical Association (vote no. 181/19).

### Subjects

**Figure 1:**
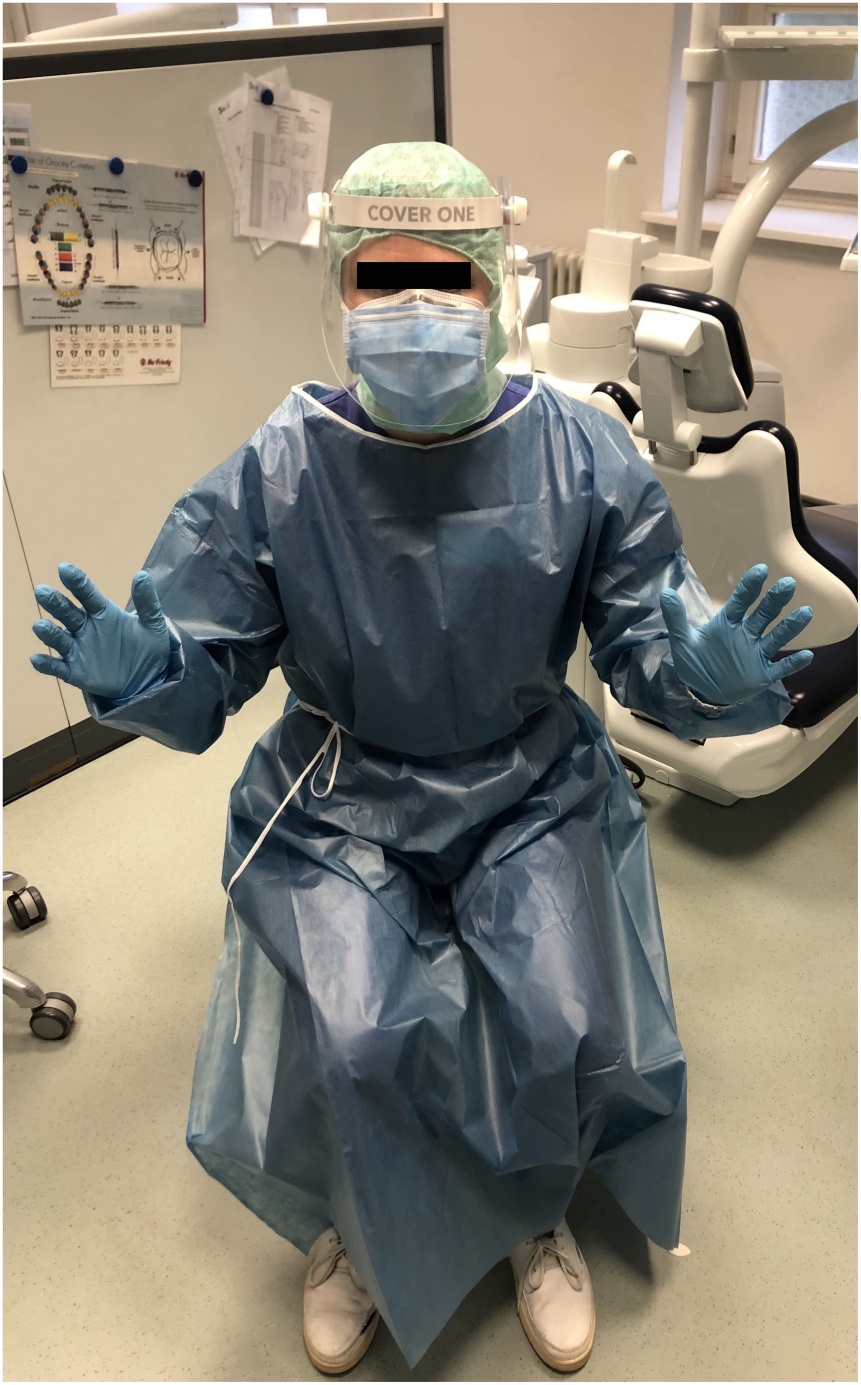
Personal protective equipment: surgeons hood, surgical mask (type 2, 3 layers), (magnification glasses), face shield, protective gown, examination gloves. Underneath the surgical face mask, a FFP-2 mask was placed.

A total of forty specifically instructed and supervised students in their 2nd and 3rd clinical year participated as study subjects. Hygienic hand disinfection was executed before the PPE was applied. During dental treatment, they wore non-sterile, clean examination gloves (nitrile powder-free gloves: Joza®, Hebei Titans Hongsen Medical Technology Co., Ltd., Hebei, China), surgical masks (tie-band medical surgical mask type II, Mölnlycke Health Care, Düsseldorf, Germany), FFP2 masks under the surgical masks (Particle Filtering Half Mask, Shunmei Medical Co., LTD., Shenzhen, China), and a face shield (Clever One, Clever Frame Baden-Württemberg, Burladingen, Germany) over the surgical masks. Furthermore, hair caps and a protective gown were worn (Figure 1). All study subjects were instructed not to touch the outer surface of their surgical mask during treatment. Mask and face shield were never in contact.

### Sampling

Samples were taken after treatment was completed. Surgical masks were removed by J. N. wearing clean examination gloves without touching the exterior surface. All surgical masks were imprinted immediately on universal media for the isolation and cultivation of microbiological samples: TSA (Trypticase^TM^ Soy Agar II with 5 % Sheep Blood, Becton Dickinson GmbH, Heidelberg, Germany; aerobic cultures) and Columbia (Columbia III Agar with 5 % Sheep Blood, Becton Dickinson GmbH, Heidelberg, Germany; anaerobic cultures) agar plates for 10 s each.

### Controls

As negative controls, five unused surgical masks (*n* = 5) were worn each for 120 min during simulated aerosol-releasing dental work on a phantom simulator.

### Microbial Cultivation

For anaerobic cultivation, Columbia agar plates were placed in a rectangular jar, a gas bag (GasPak^TM^ EZ Anaerobe Container System with Indicator, Becton Dickinson GmbH, Heidelberg, Germany) was inserted, and the container was sealed. Thereby, an oxygen concentration of less than 1% and a carbon dioxide concentration of at least 13% within 24 h are achieved. No further measures were necessary for the aerobic cultivation of the TSA plates. Both were incubated for 48 h at 35 +/- 2°C.

### Qualitative Bacterial Analysis

**Figure 2:**
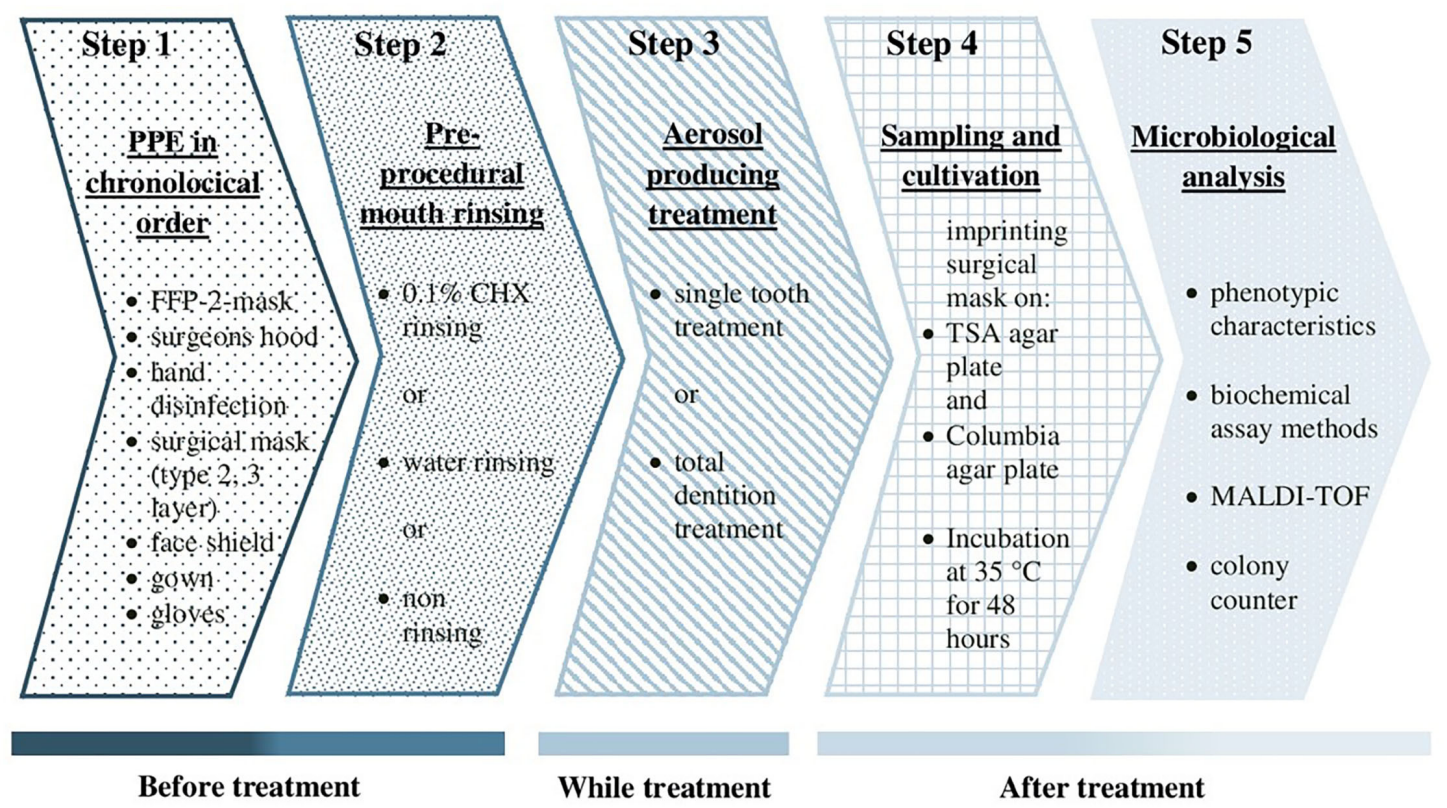
Flow chart of the study. PPE, personal protective equipment; CHX, chlorhexidine; TSA, trypticase soy agar; MALDI-TOF MS, matrix-assisted laser desorption/ ionization time of flight mass spectrometry.

The bacterial analysis was performed by phenotypic characteristics and biochemical assay methods. Colony-forming units (CFU) with clearly identical phenotypic characteristics and/or the same biochemical reactions were assumed to represent the identical genus. Colonies not being identified with certainty were examined by matrix-assisted laser desorption/ionization time of flight mass spectrometry (MALDI-TOF MS) (MBT^TM^ smart, Bruker Daltonik GmbH, Bremen, Germany) (Figure 2).

### Quantitative Bacterial Analysis

Colonies were counted using a colony counter (Schuett-biotec GmbH, Göttingen, Germany).

### Statistics

The qualitative and quantitative results of the bacterial mask contamination were presented descriptively. The mean colony numbers of samples from all three groups (CHX, water, and non-rinsing) were primarily compared with the Kruskal–Wallis (K-W) test and, in case of statistically significant differences, pairwise with the Mann–Whitney *U* test (*p* <0.05).

## Results

### Quantitative Analysis of Bacterial Contamination

**Figure 3:**
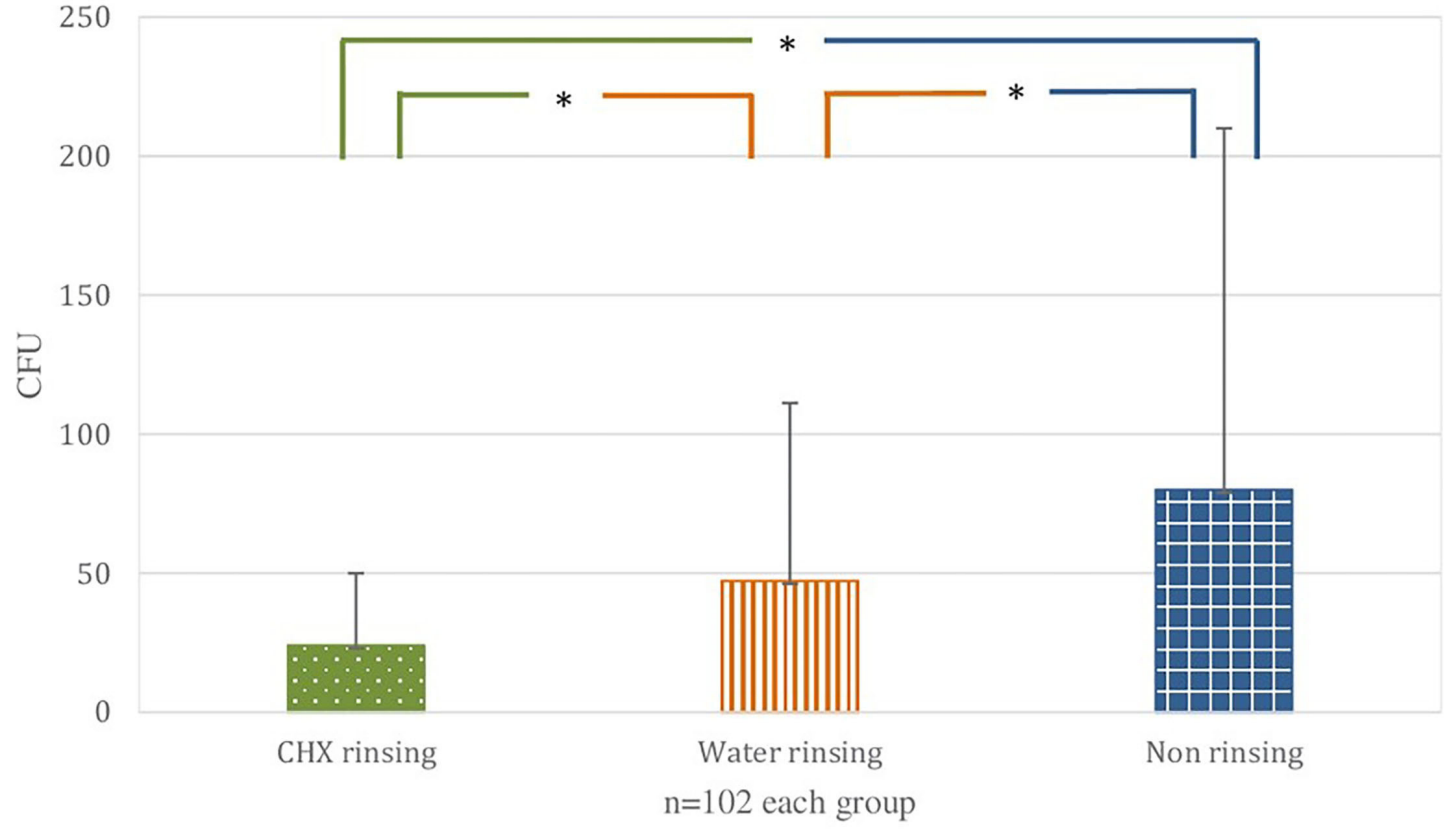
Overall comparison. Bacterial load (colony forming units, CFU) on the surgical mask behind face shield in the three groups. The bars correspond to the mean value, the line on top to the ± standard deviation. Statistically significant differences (*p* < 0.05) are marked with an asterisk.

The overall mean bacterial load on the surgical mask was lowest with 24 CFU ± 26 in the CHX group, increased with 47 CFU ± 64 in the water group, and highest with 80 CFU ± 130 in the non-rinsing group. The comparison of all groups showed a statistically significant difference (K-W test: *p* <0.00001). CHX led to a significant contamination reduction in comparison with water (*U* test: *p* = 0.003) and non-rinsing (*U* test: *p* < 0.0001). Furthermore, CFU values for water were significantly lower (U test: *p* = 0.029) in comparison with the non-rinsing group (Figure 3).

No statistically significant differences (K-W test: *p* > 0.05) could be determined between the single-tooth and total dentition treatments.

In the single-tooth treatment, CHX (mean value: 24 CFU ± 27) and water (mean value: 45 CFU ± 68) led to a statistically significant (K-W test: *p* <0.05) reduction of bacteria on masks compared to the non-rinsing group (mean value: 80 CFU ± 78). No statistically significant difference was found between CHX and water-rinsing groups (K-W test: *p* < 0.05).

**Figure 4:**
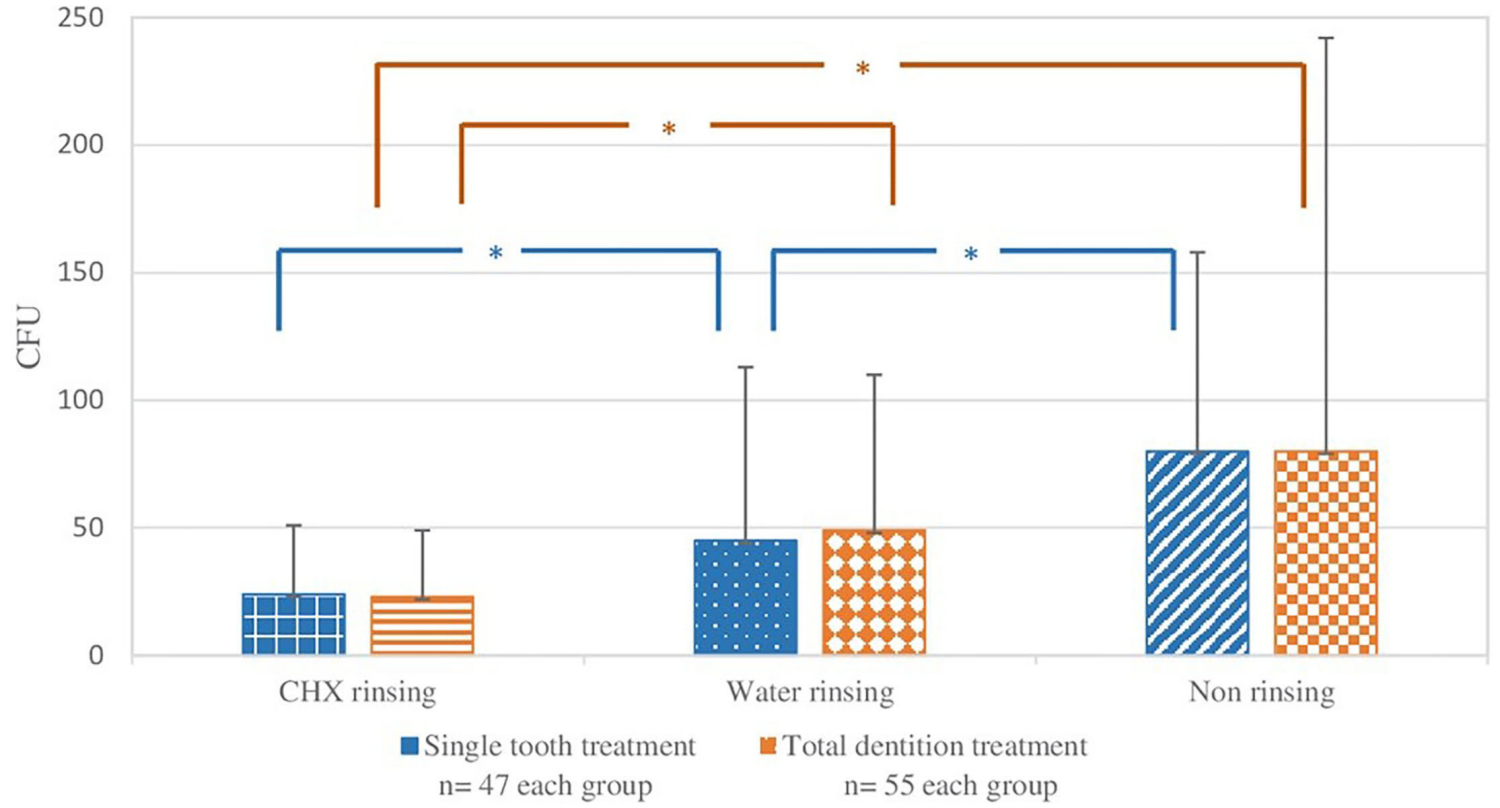
Single-tooth and total dentition treatments. Bacterial load (colony-forming units, CFU) on the surgical mask behind the face shield in the three groups. The bars correspond to the mean value, the line on top to the ± standard deviation. Statistical significance (*p* < 0.05) is marked with an asterisk.

For total dentition interventions, the comparison of all groups showed a statistically significant difference (K-W test: *p* = 0.0002), CHX (mean value: 23 CFU ± 26) led to a statistically significant bacterial reduction compared to water (mean value: 49 CFU ± 61, U test: *p* = 0.001) and the non-rinsing group (mean value: 80 CFU ± 162, U test: *p* = 0.0001). The water group was statistically not significantly different (*p* < 0.05) compared to the non-rinsing group (Figure 4).

### Qualitative Analysis of Bacterial Contamination

*Streptococcus* spp., *Staphylococcus* spp., *Micrococcus* spp., and *Bacillus* spp., representing the oral and cutaneous flora, dominated. The prevalence of *Staphylococcus aureus* was highest in the non-rinsing group (12), followed by the water group (7), and lowest in the CHX group (5) (Table 1).

**Table 1:** Detection frequency of bacterial species for CHX rinsing, water rinsing and, non-rinsing (102 samples each).

**Bacteria**	**CHX** **group**	**Water** **group**	**Non-rinsing** **group**
*Actinomyces naeslundii*	-	-	1
*Advenella incenata*	-	1	-
*Bacillus spp*.	26	35	32
*B. amyloliquefaciens*	-	-	1
*B. cereus*	1	1	1
*B. circulans*	1	1	-
*B. clausii*	1	-	-
*B. flexus*	2	2	1
*B. horneckiae*	-	-	1
*B. licheniformis*	-	-	2
*B. megaterium*	-	-	1
*B. psychrosaccharolyticus*	1	-	-
*B. pumilus*	-	-	2
*B. oceanisediminis*	1	1	-
*B. simplex*	-	-	1
*B. thermoamylovorans*	-	-	1
*B. thuringiensis*	-	2	1
*B. zeae*	-	-	1
*Brevibacillus parabrevis*	-	1	-
*Brevibacterium casei*	-	1	-
*Chryseobacterium shandongense*	-	1	-
*Clostridium perfringens*	1	-	-
*Coagulase-negative staphylococci*	81	71	74
*Cutibacterium acnes*	-	-	2
*Enterococcus spp*.	1	-	-
*Gram-negative bacteria*	-	1	2
*Kocuria rhizophila*	-	2	-
*Lactococcus lactis*	-	-	1
*Lysinibacillus sphaericus*	-	-	1
*Micrococcus spp*.	24	27	30
*Micrococcus luteus*	15	12	11
*Moraxella spp*.	-	1	-
*Neisseria spp*.	-	2	-
*Neisseria macacae*	-	1	-
*Neisseria subflava*	-	1	1
*Paenibacillus spp*.	-	1	-
*Paracoccus yeei*	1	-	-
*Propionibacterium spp*.	-	-	1
*Pseudoarthrobacter chlorophenolicus*	-	1	-
*Psuchrobacillus psuchrodurans*	-	-	1
*Rothia aeria*	-	1	-
*Rothia dentocariosa*	-	-	1
*Rothia mucilaginosa*	-	-	1
*Staphylococcus aureus*	3	5	10
*S. auricularis*	-	1	-
*S. epidermidis*	14	20	24
*S. capitis*	13	21	16
*S. haemolyticus*	2	1	1
*S. hominis*	5	8	7
*S. saprophyticus*	1	2	-
*S. succinus*	1	-	-
*S. warneri*	1	1	1
*Streptococcus spp., α-hemolysis*	11	7	8
*Streptococcus spp., γ-hemolysis*	2	17	9
*S. constellatus*	-	-	1
*S. cristatus*	1	-	-
*S. gordonii*	3	1	2
*S. mitis*	1	1	1
*S. oralis*	2	1	3
*S. parasanguinis*	1	-	-
*S. sanguinis*	-	1	2
*Veillonella parvula*	-	-	2

### Controls

All control samples displayed no bacterial growth.

## Discussion

To our best knowledge, this is the first study to investigate the effect of a pre-procedural mouth rinse in combination with a face shield on contamination of surgical masks after aerosol-producing dental treatments. Since COVID-19 pandemic started, face protection is recommended to prevent infection by droplets, splashes, or bioaerosols (13, 14). While the distribution of flashes on face shields is already investigated pointing out the highest contaminated areas are the inner part of the eyes and around the nose (15), it remains unclear to what extent areas of the face are contaminated by bioaerosol despite face shield.

At least, in an aerosol model experiment with fluorescent dye, Bentley et al. (16) showed contamination of a single-layered mask despite wearing a face shield, which is in line with our results. Even on nose and interior surface of the mask, the fluorescent dye could be found. Basically, there is less available evidence on the protective effect of face shields. Current studies indicate combining the mask and face shield may offer the best protection against bioaerosols (17). Unfortunately, due to heterogeneity of localizations, methods, and cultivation of samples, a comparison of CFUs with other studies and further discussion at this point in time is not possible. Therefore, we currently conclude that face shields are not offering complete protection against bioaerosols. However, protection against direct splashes and droplets is likely and the combination of mask and face shield may currently offer the most protection. Further research is urgently needed.

Chlorhexidine is a broad-spectrum antiseptic effective against Gram-positive and negative bacteria, furthermore fungi. It is used in dentistry to control dental biofilms' growth and treat periodontitis or root canals, especially *Enterococcus faecalis* (18, 19). At the latest since the COVID-19 pandemic, a pre-procedural mouth rinse with CHX is widely accepted in dentistry, although an antiviral effect in the oral cavity was unproven so far (20). In the meantime, studies indicating a temporarily clinical effect of a CHX mouth rinse against COVID-19 are published (21, 22).

Chlorhexidine as a pre-procedural mouthwash is effective in reducing contamination of bioaerosols at various concentrations (23). Furthermore, *in vitro* studies showed SARS-CoV-2 suppression after using 0.12% CHX (15 ml) for 2 h (18).

Most studies in this context, however, focused on bioaerosol contamination during periodontal treatment, testing a pre-procedural mouth rinse with mainly CHX, but also CPC. A review from 2021 summarized a pre-procedural rinsing for 30 s to 2 min with antimicrobial solutions, especially CHX, reduces bioaerosol contamination in periodontal prophylaxis effectively in comparison with water and non-rinsing. The results are in line with our findings (23). Also, described and confirming our results in the literature is a certain antimicrobial effect of a pre-procedural water rinse (6). Our results demonstrate the antimicrobial effect of water as a pre-procedural mouth rinse in comparison with the non-rinsing group, yet it is clearly inferior to CHX.

The results of this study confirm earlier studies by our research group (3). Masks are regularly contaminated during aerosol-producing dental treatments.

The statistical analysis showed significant differences between all three groups (CHX-, water-, and non-rinsing group), but not in all subgroups (total dentition and single-tooth treatments). We attribute this to underpowering in the subgroups, which were not foreseen in experimental design and only collected for fine analysis and presentation of the data.

Almost, all masks were contaminated with bacteria of the dermal and oral microbiome with a large diversity of bacterial species.

*Staphylococcus aureus* being lower in all three groups than reported in the literature (24), most frequently was found in the non-rinsing group (three times as high as with CHX rinse), showing pre-procedural mouth rinse effectively reduces the contamination with *S. aureus* on the mask, but even with a face shield contamination cannot be prevented. Because treatments were performed in the student course, *S. aureus* was expected to be higher (25, 26), although lower carriage rates are also described (27). Possibly, maximum protective measures and precautions since the COVID-19 pandemic started are reasons. Furthermore, only patients without general diseases participated in the study, and dental staff was informed and highly compliant with the hygienic standards in our clinic. In any case, *S. aureus* is a potentially high-risk, multiresistant, nosocomial pathogen, and its transmission to the mask cannot be prevented by the highest protective measures.

Some coagulase-negative staphylococci are potentially high-risk, multiresistant, nosocomial pathogens, for example, *Staphylococcus epidermidis* (28, 29).

Other microorganisms, for example, *Staphylococcus capitis* and *Streptococcus oralis* and other bacteria are part of the commensal microbiome. To healthy individuals, these microorganisms are not posing a threat, but may do to immunosuppressed or immunocompromised patients. The patient's health status and the risk factors for a facultative pathogenic species to become a pathogenic one are not always obvious, and it is important to implementing constant compliance with regulations and recommendations for the prevention of nosocomial infections (30). Furthermore, a patient's colonization with pathogenic or facultative pathogenic bacteria is not possible to assess, and transmission to a susceptible patient or even dental healthcare professional cannot be excluded. For transmission, infection and clinical manifestation of diseases frequency of exposure and the virulence of the pathogen are important (31).

Basically, no obligate pathogens could be detected on the mask. Only healthy patients without known infectious diseases or without immunosuppression were included in the study. Therefore, pathogens are underrepresented by patient selection, and the results do not indicate that pathogens cannot be transmitted from the mask. Moreover, the basic route of contamination has already been demonstrated by our research group (3).

The methodology used in this study had strengths and limitations. Only viable bacteria will be detected on agar. Using a different method (e.g. nucleic acid-based methodes) would probably have led to a larger number of detected bacteria, showing the potential of aerosols to transport viable and dead bacteria. Nevertheless, only viable bacteria can be transferred, posing a potential transmission and infection risk. Usually, the agar used detects fast-growing bacteria, probably underestimating slow-growing colonies. Competitive growth may have reduced the number of bacteria, nevertheless, only easily cultivable bacteria are important when investigating transmissions. The sample collection may lead to underestimation of microbial diversity and contamination on the masks. First, agar only detects pathogens adhering to the mask surface. Second, the mask surface cannot completely be brought in contact with the agar plate. The MALDI-TOF MS analysis we used is restricted to colonies identified as different phenotypes potentially resulting in an underestimation of the bacterial spectrum. In summary, the total number of viable bacteria on the mask must be assumed to be actually higher.

In our study, contamination of the surgical mask and thus transmission of droplets and bioaerosols into the facial region could not be completely prevented through a face shield and a pre-procedural mouth rinse. This is remarkable, especially in times of a pandemic, when it is important to correctly assess the effect of the PPE or mechanisms of infection control and not to live in a false sense of security. The contamination by viruses and the effect of a pre-procedural mouth rinse on the contamination load of the surgical mask were not investigated representing an additional risk for patients and dental staff. Obviously, there are unknown transmission paths past the face shield. A comparison of a group of students wearing a face shield with a group not doing it was not possible due to the strong general rules of infection control. The increasingly discussed emergence of CHX-resistant bacteria may be another reason for the contamination detected (32, 33). Nevertheless, mask contamination was clearly reduced by a CHX mouth rinse, and during COVID-19 pandemic, at least any additional bacterial transmission or possible infection must be prevented.

## Conclusion

A pre-procedural mouth rinsing reduces the contamination load on the surgical mask. However, even with an additional face shield, contamination of the mask cannot be prevented, so neither offers complete protection. Specifically, in view of the COVID-19 pandemic, it is crucial knowing complete covering of the face with a face shield, and an additional rinsing with CHX is not leading to a complete reduction of transmission of aerosols and droplets into the facial region. Nevertheless, mask contamination was clearly reduced by a CHX mouth rinse. During COVID-19 pandemic, at least any additional bacterial transmission or possible infection must be prevented. A pre-procedural mouth rinsing with water also reduces the contamination load. It should be used if antiseptic rinsing solutions are not available. In the future and depending on the pandemic situation, studies should be set up investigating the research question with and without a face shield.

## Data Availability Statement

The original contributions presented in the study are included in the article/supplementary material, further inquiries can be directed to the corresponding author.

## Ethics Statement

The studies involving human participants were reviewed and approved by Ethics Committee of the Saarland Medical Association. Written informed consent for participation was obtained from the study participants (healthcare practitioners) to take part in this study.

## Author Contributions

MG, JN, GB, MH, BG, AH, and SR planned the study, analyzed, interpreted the data, and were the major contributors to writing the manuscript. JN conducted the study. JN and MG supervised the study. All authors gave final approval and agreed to be accountable for all aspects of the work.

## Conflict of Interest

The authors declare that the research was conducted in the absence of any commercial or financial relationships that could be construed as a potential conflict of interest.

## Publisher's Note

All claims expressed in this article are solely those of the authors and do not necessarily represent those of their affiliated organizations, or those of the publisher, the editors and the reviewers. Any product that may be evaluated in this article, or claim that may be made by its manufacturer, is not guaranteed or endorsed by the publisher.
